# Overexpression of the Aryl Hydrocarbon Receptor (Ahr) Mediates an Oxidative Stress Response following Injection of Fine Particulate Matter in the Temporal Cortex

**DOI:** 10.1155/2020/6879738

**Published:** 2020-12-28

**Authors:** So Young Kim, Kyung Woon Kim, So Min Lee, Da-hye Lee, Sohyeon Park, Bu Soon Son, Moo Kyun Park

**Affiliations:** ^1^Department of Otorhinolaryngology, CHA University College of Medicine, Seongnam, Republic of Korea; ^2^Department of Medical Biotechnology, SoonChunHyang University, Asan, Chungnam, Republic of Korea; ^3^Department of Otorhinolaryngology, Seoul National University College of Medicine, Seoul, Republic of Korea; ^4^Sensory Organ Research Institute, Seoul National University Medical Research Center, Seoul, Republic of Korea

## Abstract

Studies have shown that particulate matter (PM) induces the expression of the aryl hydrocarbon receptor (Ahr) leading to the activation of the oxidative stress response. This study is aimed at characterizing the specific impact of fine PM on the expression profile of the Ahr and oxidative stress response in the primary auditory cortex. PM_2.5_ (<1.8 *μ*m)-loaded filters were suspended in sterile saline to 102.6–111.82 *μ*g/ml. Next, 10 *μ*l of PM_2.5_ or an equal volume of saline was administered intracranially into the temporal cortex of two groups of rats (PM_2.5_ and control; *n* = 14 per group), respectively. One week after intracranial injection, the temporal cortex was harvested. Transmission electron microscopy was performed to evaluate the distribution of PM_2.5_ within the temporal cortex. Additionally, the mRNA and protein expression levels of cytochrome P450 1A1 (*CYP1A1*), *CYP1B1*, inducible nitric oxide synthase (*iNOS*), Ahr, and brevican mRNA and protein were measured using quantitative reverse transcription-polymerase chain reaction (qRT-PCR) or western blotting, respectively. Finally, the protein expression levels of the receptor for advanced glycation end products (RAGE) were estimated using enzyme-linked immunosorbent assay (ELISA). PM_2.5_ was observed in intracellular vesicles within the temporal cortex following intracranial injection. Levels of oxidative stress molecules (i.e., *CYP1A1*, *CYP1B1*, and *iNOS*), Ahr, Brevican, and RAGE were higher in the PM_2.5_ group compared with the control group. Intracranial administration of PM_2.5_ led to increased levels of Ahr and markers of an oxidative stress response in the temporal cortex. The oxidative stress response-mediated increases in the levels of brevican and RAGE.

## 1. Introduction

Particulate matter (PM) has been suggested to have adverse effects on the central nervous, respiratory, and cardiovascular systems. In a clinical study involving subjects from the United States, long-term exposure to PM_2.5_ (fine particles < 2.5 *μ*m) and PM_10_ (inhalable particles < 10 *μ*m) was associated with reduced brain volume [1]. A number of other studies have described CNS injuries after inhalation of PMs [2–5]. In a rat study, long-term (i.e., 3 and 6 months) exposure to PM_1_ induced a short-term memory deficit, spongiosis, and neuronal shrinkage and led to increases in proinflammatory molecules including interleukin (IL) 6 in the hippocampus [2]. In a mouse inhalation study, inhalation of ambient PM for 4 weeks led to (i) an upregulation of proinflammatory molecules, (ii) downregulation of molecules of the brain-derived neurotrophic factor (BDNF) signaling pathway (e.g., BDNF, high-affinity receptor tropomyosin-related kinase B (TrkB), cyclic adenosine monophosphate-response element-binding protein), and (iii) depressive-like behavioural changes [3]. Previous studies by this group have also demonstrated proinflammatory and oxidative stress responses, alterations in the expression of vesicular synaptic transporters, and perineuronal changes following 4-week and 8-week exposures to PM_10_ in cerebral cortical areas [4, 5].

Systemic cardiovascular circulation and inhalation through the olfactory bulb cleft might be a primary route for PMs to reach the CNS in inhalation models [6]. Cerebral blood circulation might preferentially lead to an effect on brain regions with high metabolic rates; therefore, areas like the cortex might be more susceptible to chronic DEP inhalation-induced oxidative stress and inflammation. In line with this, previous animal inhalation studies reported more cerebral cortical inflammation and oxidative stress injuries compared with olfactory bulb injuries [3–5]. Relating to the diffusion of inhaled PMs via the nasal olfactory system, a number of previous studies reported olfactory deposition and translocation to the olfactory bulb [7–10]. Direct PM exposures such as those described above could lead to penetration and destruction of cellular integrity [11]. An *in vitro* study described the reduction of cellular integrity by transepithelial electric resistance following 24-hour exposure to commercial cigarette smoke condensates in brain tissue and astrocytes in the presence of a blood-brain barrier [11]. However, the direct penetrating effect of PM on the CNS has not been explored *in vivo*.

The aryl hydrocarbon receptor (AhR) has been proposed as a signal transducer of PM-induced oxidative stress and inflammation [12, 13]. *AhR* is a basic helix-loop-helix PER/ARNT/SIM family of transcription factors, which is activated by several exogenous ligands (e.g., polycyclic aromatic hydrocarbons and halogenated aromatic hydrocarbons) [14]. When *AhR* ligands activate the *AhR* and Aryl hydrocarbon receptor nuclear translocator, they bind to consensus xenobiotic responsive elements (XRE) located upstream of target genes (e.g., cytochromes P450 such as *CYP1A1*) [15]. Several published studies have reported *AhR* activation in PM-induced respiratory or cardiovascular diseases [16, 17]. However, the molecular mechanisms of PM-mediated *AhR* activation in the CNS have not been well characterized.

This study hypothesized that the direct effects of PM_2.5_ on the cerebral cortex might be accompanied by the intracellular translocation of PM_2.5_ and that the proinflammatory and oxidative stress responses could be mediated by increases in *AhR*. To investigate the direct impact of PM_2.5_ on the temporal cortex *in vivo*, PM_2.5_ was injected intracranially in adult rats. The presence of PM_2.5_ in the temporal cortex was confirmed, and downstream response genes of *CYP1A1* and *CYP1B1* and inflammatory molecules were evaluated.

## 2. Methods

### 2.1. Animal Experiments

**Figure 1:**
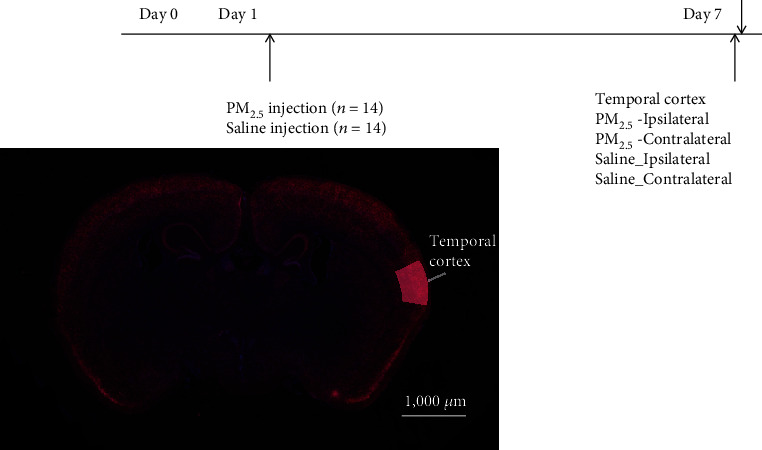
The experimental schedule of the present study. Postnatal 8-week-old female Sprague-Dawley rats received intracranial injections of PM_2.5_ (particulate matter diameter < 1.8 *μ*m) or saline in the primary temporal cortex. The injection site of the temporal cortex was indicated with a red arrow.

The study was approved by the Institutional Animal Care and Use Committee of CHA University Medical School (IACUC170162). Postnatal 8-week-old 10 female and 4 male Sprague-Dawley rats were divided into PM_2.5_ and saline groups (*n* = 14 per group) (Figure 1). The PM_2.5_ rats received intracranial injections of 10 *μ*l of 102.6–111.82 *μ*g/ml PM_2.5_ (diameter < 1.8 *μ*m) in the primary temporal cortex. Under anesthesia using a mixture of Zoletil (40 mg/kg) and xylazine (10 mg/kg), a 10 *μ*l 33-gauge needle Hamilton syringe was used for the intracranial injections with a stereotactic apparatus as previously described [18]. The injection site was as follows (in mm from bregma and the skull): anterior posterior (AP): -5 mm, lateral (L): 7 mm, ventral (V): 4.2 mm according to the atlas of Paxinos and Watson (2006). After the cortical bone was drilled, the meninges were punched with a sterile needle. The total injection volume of 10 *μ*l was infused with a speed of 0.2 *μ*l/min using an automated microinjector (Harvard Apparatus, Saint-Laurent, QC, Canada), and the injected needle was maintained for 50 minutes [19–21]. In the saline group (*n* = 10), 10 *μ*l of saline was similarly injected into the primary temporal cortex. No rats died after intracranial injection, and no differences in weight and activities between PM_2.5_ and control groups were observed. All rats were sacrificed, and ipsilateral (intracranial injected site, right) and contralateral (left) temporal cortices were harvested as frozen tissue in 18 rats (*n* = 9 for each group). Whole brain tissue was harvested from the remaining 6 rats for histologic examination and immersion-fixed in 4% paraformaldehyde solution.

### 2.2. Fine Particulate Matters

Fine particulate matters were collected in an industrial region (Asan, Korea) five times between September 15, 2017, and November 21, 2017, using a previously described process [22]. The collected airborne fine particles were filtered on Teflon (PTFE) filters (Whatman, Maidstone, Kint, UK). After drying the filters in a desiccator for 48 hours, the weights of PM_2.5_ were measured using a microbalance (CP2P-F, Satorius, Coettingen, Germany). After baking for 2 hours, the filters were immersed in deionized water and sonicated for 1 hour. The compositions of heavy metals in PM_2.5_ were analyzed and are presented in Table 1.

### 2.3. Transmission Electron Microscopy

To characterize the localization of the injected PM_2.5_ in the temporal cortex, brain sections of PM_2.5_ rats were examined using transmission electron microscopy (TEM) [23]. The immersion-fixed brain samples were washed using deuterated H_2_O_2_ and dehydrated using ethanol solutions. The samples were infiltrated by propylene oxide and EPON epoxy resin mixed (Embed 812, Nadic methyl anhydride, poly Bed 812, dodecenylsuccinic anhydride, and dimethylaminomethyl phenol) (Electron Microscopy Polysciences, USA). The epoxy resin-embedded brains were polymerized at 38°C for 12 hours and 60°C for 48 hours. Using an ultramicrotome (RMC MT-XL), samples were thinly sectioned (roughly 65 nm). Sections were then stained with saturated 4% uranyl acetate and 4% lead citrate. Finally, tissues were examined by TEM (JEM-1400, Japan) at 80 kV.

### 2.4. Expression Levels of Cytochrome P450 (CYP) Enzymes and Aryl Hydrocarbon Receptor (AhR)

Real-time reverse transcription-polymerase chain reaction (RT-PCR) was performed using micropunched primary temporal cortex tissue as previously detailed [4]. Reverse transcription was performed using TOPreal™ qPCR 2X PreMIX' (Enzynomics, #RT500M) and forward and reverse oligonucleotides for PCR amplification of cytochrome P450 (CYP)1A1, CYP1B1, inducible nitric oxide synthase (iNOS), and AhR (Table 2) using CFX connect™ Real-time system (Bio-Rad, #1855201). mRNA levels were normalized to glyceraldehyde 3-phosphate dehydrogenase.

### 2.5. Changes in the Expression Levels of AhR Protein in the Primary Temporal Cortex

Immunofluorescence staining was performed as previously described [4]. Specimens were dehydrated and embedded in paraffin with optimal cutting temperature solution. The 10 *μ*m sections of embedded tissue were cut on a rotary microtome and mounted on glass slides. Each slide was dipped in xylene for paraffin removal for 10 min and sequentially washed in 100%, 75%, and 50% ethanol for 5 min each wash. The free-floating sections were then washed in PBS three times for 5 min per wash. After a set of three 5 min washes in PBS, sections were placed in 10% goat or donkey blocking serum (Vector Labs, Burlingame, CA, USA) for 1 hour at room temperature. Free-floating slices were then incubated overnight at 4°C on a shaking table with an anti-Ahr primary antibody (mouse monoclonal, Santa Cruz Biotechnology). The following day, slices were washed in PBS three times for 10 min per wash. Then, sections were incubated with secondary antibody (Alexa Fluor 594 donkey anti-mouse (Invitrogen, USA, #A21203)) for 2 hours at room temperature. The primary auditory cortex (A1) areas from each hemisphere were analyzed: AP: -5 mm, L: 7 mm, and V: 3.1 – 4.9 mm. Images were photographed using a fluorescence microscope (ECLIPSE Ni-U, Nikon Corporation, Japan).

### 2.6. Protein Expression Levels of Matrix Metalloproteinase (MMP) 9 and Brevican

Western blotting was performed as previously described [4]. Approximately 20 *μ*g of protein was separated by 12% sodium dodecyl sulphate-polyacrylamide gel electrophoresis (SDS-PAGE) and transferred to polyvinylidene difluoride (PVDF) membranes (Merck Millipore, Burlington, MA, USA). Membranes were soaked in blocking buffer (5% nonfat dry milk in Tris-buffered saline containing Tween-20 [TBS-T]) for 1 hour at room temperature followed by incubation with specific primary antibodies: anti-MMP9 (rabbit monoclonal, Abcam), antibrevican (rabbit polyclonal, Abcam), and *β*-actin (D6A8, rabbit mAb; Cell Signaling Technology). Immunoreactive proteins were detected with a horseradish peroxidase- (HRP-) coupled secondary antibody (anti-rabbit IgG, HRP-linked antibody; Cell Signaling Technology) and visualised using an enhanced chemiluminescence (ECL) kit (Bio-Rad). Protein bands were quantitated by densitometry using ImageJ gel analysis software (National Institutes of Health, Bethesda, MD, USA). The protein expression levels were normalized to *β*-actin.

### 2.7. Protein Expression Level of RAGE

The levels of RAGE protein were determined by enzyme-linked immunosorbent assay (ELISA). We used the corresponding quantitative rat Immunoassay ELISA (ab202409, Abcam) according to the manufacturer's instructions. Briefly, absorbance was measured spectrophotometrically at 450 nm (SpectraMax M2, Molecular Devices, USA). The temporal cortex was homogenized with PBS (pH 7.4) solution containing 1% Triton X-100 and then centrifuged at 3000 rpm for 15 min. The supernatant was collected and immediately stored at −80°C until use.

### 2.8. Statistical Analysis

Statistical analyses were performed using the *T*-test after testing for normality using the Shapiro-Wilk test. Values are expressed as means ± standard deviation. SPSS software (ver. 21.0; IBM Corp., Armonk, NY, USA) was used for all analyses, and statistical significance was defined as *P* < 0.05.

## 3. Results

**Figure 2:**
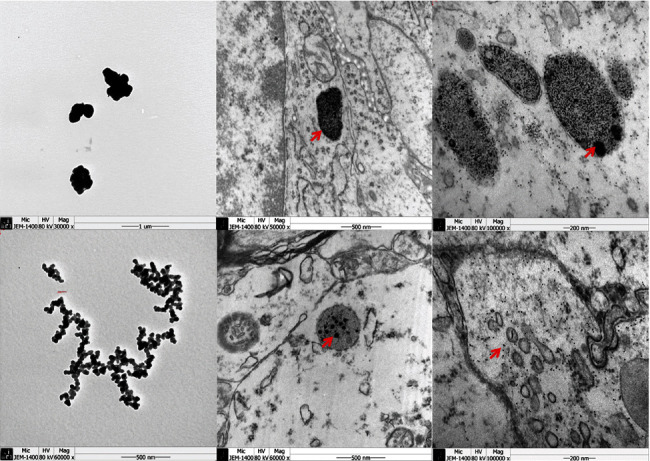
Transmission electron microscopy examination of injected brain tissue. The electron-dense particles (red arrow) accumulated in vesicles within the cells of the brain in rats from the PM_2.5_ group.

TEM demonstrated that the electron-dense particles accumulated in vesicles within the cells of multiple areas of the brain in rats from the PM_2.5_ group, an observation which suggests endocytosis of PM_2.5_ (Figure 2).

**Figure 3:**
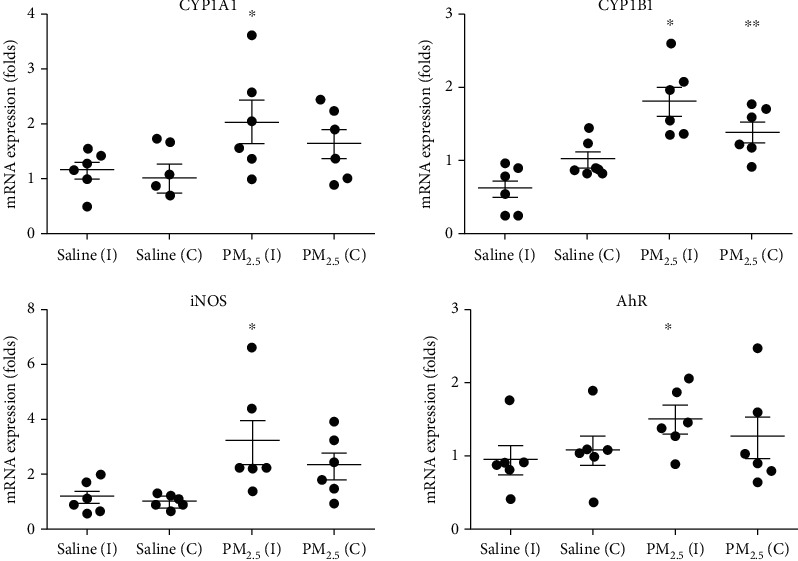
Comparisons of the mRNA expression levels of cytochrome P450 1A1 (*CYP1A1*), *CYP1B1*, inducible nitric oxide synthase (*iNOS)*, and aryl hydrocarbon receptor (*Ahr)* between saline and PM_2.5_ groups. The PM_2.5_ group showed higher mRNA expression levels of *CYP1A1*, *CYP1B1*, *iNOS*, and *Ahr* in the ipsilateral side of PM_2.5_ injections. (^∗^*P* < 0.05*T*-test between ipsilateral saline vs. ipsilateral PM_2.5,_^∗∗^*P* < 0.05*T*-test between contralateral saline vs. contralateral PM_2.5,_ I: ipsilateral; C: contralateral).

The mRNA expression levels of *CYP1A1*, *CYP1B1*, *iNOS,* and *Ahr* were higher in the PM_2.5_ group compared with the saline group (Figure 3). The mRNA levels of *CYP1A1* in the ipsilateral temporal cortex in the PM_2.5_ group were 2.02 (SD = 0.95) fold higher compared with those in the saline group (*P* = 0.05 in *T*-test). The levels of *CYP1A1* mRNA in the contralateral temporal cortex in the PM_2.5_ group were not significantly different from those in the saline group (*P* = 0.126 in *T*-test). The levels of *CYP1B1* mRNA in the ipsilateral and contralateral temporal cortices in the PM_2.5_ group were 1.81 (SD = 0.49) and 1.38 (SD = 0.33) fold higher than those in the saline group, respectively (*P* < 0.001 and *P* = 0.002 in *T*-test). Levels of *iNOS* mRNA in the ipsilateral and contralateral temporal cortices in the PM_2.5_ group were 3.15 (SD = 1.98) and 2.29 (SD = 1.12) fold higher compared with those in the saline group (*P* = 0.037 and *P* = 0.48 in *T*- test).

**Figure 4:**
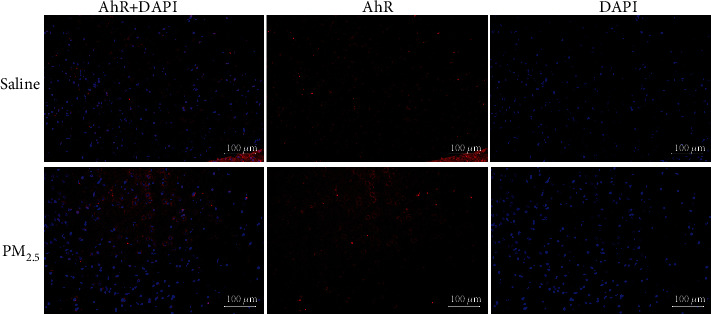
Immunofluorescence examination of AhR expressions. The levels of the AhR protein (red) in the ipsilateral primary temporal cortex were increased in the PM_2.5_ group compared to the saline group (blue: DAPI).

The mRNA levels of *AhR* in the ipsilateral temporal cortex in the PM_2.5_ group were 1.48 (SD = 0.42) fold higher compared with those in the saline group (*P* = 0.05 in *T*-test), however, they were not significantly different in the contralateral temporal cortex (*P* = 0.374 in *T*-test). Upon immunofluorescence examination, the levels of the AhR protein in the ipsilateral primary temporal cortex were increased in the PM_2.5_ group compared to the saline group (Figure 4).

**Figure 5:**
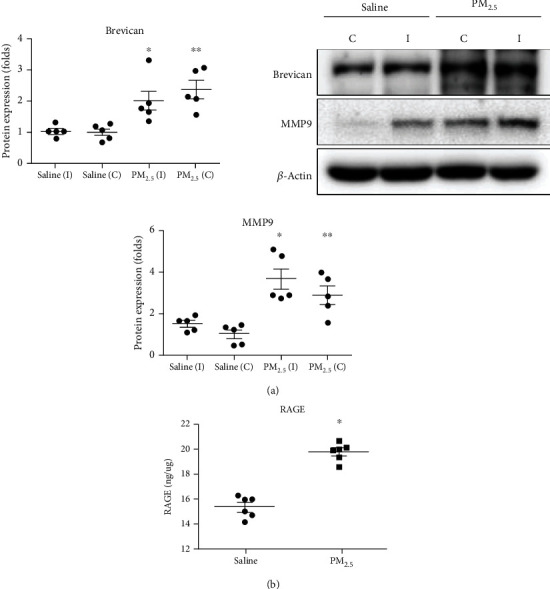
Comparisons of the protein expression levels of brevican, MMP9, and RAGE. (a) In western blotting, the PM_2.5_ group showed higher protein expression levels of brevican and MMP9 in the ipsilateral side of PM_2.5_ injections. (b) Enzyme-linked immunosorbent assay showed a higher expression level of advanced glycation end products (RAGE) in the PM_2.5_ group (^∗^*P* < 0.05*T*-test between ipsilateral saline vs. ipsilateral PM_2.5,_^∗∗^*P* < 0.05*T*-test between contralateral saline vs. contralateral PM_2.5_,_,_ I: ipsilateral; C: contralateral).

The abundance of MMP9 and brevican proteins were 2.02 (SD = 0.77; *P* = 0.02 in *T*-test) and 2.02 (SD = 0.77; *P* = 0.02 in *T*-test) fold higher in the ipsilateral temporal cortex of the PM_2.5_ group compared with the saline group, respectively (Figure 5).

Detection of RAGE protein levels using ELISA demonstrated an elevation in the PM_2.5_ group compared with the saline group. The levels of RAGE protein in the ipsilateral temporal cortex of the PM_2.5_ group were 1.29 (SD = 0.05) fold higher compared with those in the saline group (*P* < 0.001 in *T*-test).

## 4. Discussion

The intracranial injection of PM_2.5_ increased the levels of AhR in the primary temporal cortex. These increases might be linked with the increased expression levels of oxidative stress molecules (e.g., *CYP1A1*, *CYP1B1*, and *iNOS*). In addition, an increase in the abundance of AhR was accompanied by an increase in the amount of MMP9, a molecule which has been suggested to activate RAGE. These effects of PM_2.5_ were evident by direct intracellular penetration of nanoparticles as well as by indirect pathways through systemic circulation or paracrine effects.

The impact of PM_2.5_ on the temporal cortex was triggered by AhR activation. A number of previous studies have suggested that PM trigger proinflammatory reactions triggered by AhR-dependent pathway(s) [24, 25]. In a 3D culture study, which imitated the alveolar-capillary barrier, diesel exhaust particle exposure led to the upregulation of AhR-regulated genes including *CYP1B1* and proinflammatory molecules including *MMP1*; these responses were inhibited by an AhR antagonist [24]. AhR activation might be involved in the proinflammation responses within the CNS like those occurring in the respiratory system. In lipopolysaccharide-activated microglia, the expression of tumor necrosis factor-alpha, *iNOS*, and *CYP1A1* was increased and reduced following the knockdown of *AhR* expression in the cerebral cortex of mice [25]. Therefore, it could be presumed that AhR activation may have a crucial role for inducing PM_2.5_-mediated inflammatory responses in cerebral cortical areas.

In the current study, the levels of key oxidative stress molecules (i.e., *CYP1A1*, *CYP1B1*, and *iNOS*) increased following exposure to PM_2.5_. Many genes, including *CYP1A1*, *CYP1B1*, and proinflammatory genes (e.g., nuclear factor-kB), have *AhR*-response elements in their promoters; thus, *AhR* activation might promote transcription of these genes [15, 26]. *AhR* could induce an inflammatory response through these *AhR*-response gene cascades, specifically CYPs, which are important oxidizing enzymes capable of metabolizing xenobiotic compounds [27]. Thus, organic compounds found in PMs could be metabolized by CYPs and produce reactive oxygen species, which in turn may trigger inflammation [24]. In an *in vitro* study, activation of the AhR/CYP1A1 pathway in cortical neurons and glial cells was described following exposure to the xenobiotic compound Di-(2-ethylhexyl) phthalate [28]. Although increases in the levels of *CYP1A1* and *AhR* in the temporal cortex were localized to the side of injection, the expression of *CYP1B1* and *iNOS* was elevated on both sides of the temporal cortex in the PM_2.5_ group. Indirect routes of PM_2.5_ exposure (e.g., cerebrovascular or cerebrospinal fluid circulation) could mediate the increase of these oxidative markers on both sides of the temporal cortex.

Here, it is reported that a PM_2.5_-mediated increase in *AhR* levels accompanied an upregulation of MMP9, brevican, and RAGE. MMP9 is a proinflammatory molecule involved in extracellular matrix (ECM) remodeling via degradation of ECM molecules. Work by this group and others have also reported increases in the expression of MMPs following exposure to PM [5, 29, 30]. The observed increase in levels of RAGE in this and several previous studies could be linked with MMP9 upregulation [31, 32]. MMP9 is suggested to mediate an inflammatory response in the CNS by activation of RAGE [33]. In addition to MMP9, AhR activation has been shown to modulate the expression of ECM proteins [34, 35]. The disruption and remodeling of ECM could contribute to the intracellular penetration of PMs and the associated inflammatory responses and fibrosis. In a study in prostate cancer cell lines, MMP9 expression increased after aryl hydrocarbon exposure, events which were suggested to facilitate tumor invasion [36]. In bronchial epithelial cells, AhR agonists led to the upregulation of MMP2, MMP9, and MMP1 and proposed to contribute to airway remodeling in asthma and chronic obstructive pulmonary disease [37]. Chronic PM exposure altered perineuronal nets in previous studies [4, 5]. The present study demonstrated an increase of brevican expression after PM_2.5_ exposures. Brevican is a component of perineuronal nets in the CNS, structures which regulate interneuronal and synaptic plasticity [38]. Therefore, alterations in the ECM via the AhR/MMP9 pathway might also have an effect on the CNS.

Although the present study demonstrated *in vivo* effects of PM_2.5_ on the cerebral cortex, further studies to delineate the components of PM_2.5_ which exert harmful effects and the specific cell types (i.e., neurons and glia) on which these effects are conferred are warranted. Due to the limitation of loading volume for intracranial injection, this study could not delineate the dose-response relations between the exposed PM_2.5_ concentrations and the cerebral cortical changes. A few previous studies reported the dose-response associations between PM dose and the inflammatory changes in brain regions [39, 40]. In addition, the functional implications of PM_2.5_ need to be further investigated to better elucidate clinically relevant applications.

## 5. Conclusion

Following intracranial injection, PM_2.5_ accumulated in the barrier-disrupted cerebral cortex. PM_2.5_ exposure led to an increase in the levels of AhR, which mediated oxidative stress responses and RAGE-linked inflammation processes in the temporal cortex. The upregulation of MMP9 and brevican might induce ECM remodeling after PM_2.5_ exposure.

## Figures and Tables

**Table 1:** Compositions of heavy metals in fine particulate matters (PM_2.5_).

Heavy metals	Cd	As	Pb	Cr	Cu	Mn	Ni	Be
Average concentrations (pg/2.72 *μ*g)	3.17	21.42	46.73	21.49	104.53	108.06	7.29	0.00
Standard deviation	2.11	13.48	30.15	22.67	34.55	33.94	3.83	0.00

**Table 2:** Oligonucleotide primer sequences for quantitative reverse transcriptase-polymerase chain reaction.

Gene	Primer sequence (forward)	Primer sequence (reverse)	Annealing temperature (°C)	Product size (bp)	RefSeq number
*CYP1A1*	5′-CATCCCCCACAGCACCATAA-3′	5′-TTCGCTTGCCCAAACCAAAG-3′	60	212	NM_012540.2
*CYP1B1*	5′-TGCTACTCGTTTCGGTCCTG-3′	5′-CAAGGCGAGCGAAGTACAAG-3′	60	162	NM_012940.2
*iNOS*	5′-AGGCCACCTCGGATATCTCT-3′	5′-TCTCTGGGTCCTCTGGTCAA-3′	60	85	NM_012611.3
*AhR*	5′-CTCCCTCCACAGTTGGCTTTGTTTG-3′	5′-GATTCTGCGCAGTGAAGCATGTCAG-3′	60	233	NM_013149.3

## Data Availability

The raw data of experiments used to support the findings of this study are available from the corresponding author upon request.
